# Evolutionary and genotypic analyses of goose astrovirus

**DOI:** 10.1016/j.psj.2025.105511

**Published:** 2025-07-03

**Authors:** Mingxiang Li, Min Wang, Jing Wang, Hao Li, Ran Guan, Guangwen Yan, Zengwen Huang

**Affiliations:** a Xichang University, Xichang, China; b Key Laboratory of Animal Epidemic Disease Detection and Prevention in Panxi District, Xichang, China; c Academy of Agricultural Sciences of Liangshan Yi Autonomous Prefecture, Xichang, China

**Keywords:** Goose astrovirus, Phylogenetic analysis, Recombination, ORF2, Molecular epidemiology

## Abstract

Based on 183 complete genome sequences of Goose Astrovirus (GAstV), this study systematically elucidated the molecular epidemiological characteristics and evolutionary patterns of the virus. Through whole-genome phylogenetic analysis, GAstV was clearly classified into two major groups: GAstV-I (GI) and GAstV-II (GII), which were further subdivided into six distinct subgroups with significant genetic divergence. Spatiotemporal dynamic analysis revealed that the virus has spread extensively to 17 provinces in China over the past decade. Notably, 27 recombination events were identified, indicating that genetic recombination serves as a key driver of GAstV evolution. 210 specific amino acid variation sites were detected in the capsid protein, among which the characteristic amino acid deletions at positions 652-653 and 706 could serve as reliable molecular markers for the GI a and GI b subgroup. These findings not only deepen our understanding of the molecular characteristics and evolutionary mechanisms of GAstV but also provide a profound theoretical basis for establishing precise viral surveillance systems and formulating effective prevention and control strategies.

## Introduction

*Goose astrovirus* (GAstV), belonging to the genus *Avastrovirus* of the family *Astroviridae*, is a non-enveloped, single-stranded, plus-sense ribonucleic acid (RNA) (Li et al., 2024). It is the pathogen responsible for the clinical manifestations of high goose gout, characterized by severe urate deposition in the kidneys, liver, and joint cavities, with mortality rates reaching 20-50% during outbreak (Zhou et al., 2024). The genome consists of three open reading frames (ORFs), arranged in the order of ORF1a, ORF1b, and ORF2 genes. The capsid protein encoded by the ORF2 gene is located on the viral envelope within the large surface protrusions of the virus particles and plays a crucial role in the attachment of virus particles to host cell receptors (Fei et al., 2022). Therefore, the ORF2 gene is considered essential for understanding the genetic relatedness and epidemiological status of GAstV, as well as for advancing vaccine development.

Since 2016, GAstV has been identified as the primary pathogens causing gout in 1-20 day-old goslings. Based on this discovery, several studies have reported an association between GAstV infection and gout in goslings across various provinces in China (Cen et al., 2023). Currently, GAstV is divided into two groups: GAstV-I (GI) and GAstV-ⅡⅡ (GⅡⅡ) (Wang et al., 2021; Zhang et al., 2022). Studies have shown that these two groups can infect independently or co-infect, with GⅡⅡ being more prevalent than GI (Shen et al., 2022). Additionally, multiple studies have demonstrated that GAstV can cross species barriers, infecting ducks and chickens, and inducing gout symptoms (Chen et al., 2020; Wei et al., 2023). Therefore, further genomic research is crucial for a better understanding of the genetic diversity, evolutionary patterns, and host-specific adaptations of GAstV, which is essential for developing effective and targeted control strategies.

Although accumulating evidence indicates that GAstV has undergone significant genetic variation (particularly in the ORF2 protein region), current research data on the genetic evolutionary patterns of GAstV genotypes, recombination events, and the impact of amino acid mutation characteristics on the overall genetic evolution of the virus remain extremely limited. To gain a comprehensive understanding of the molecular epidemiological characteristics and genetic diversity of GAstV wild-type strains, this study systematically analyzed the genetic traits and evolutionary patterns of emerging GAstV strains, aiming to provide critical scientific insights for the development of effective prevention and control strategies against GAstV-associated diseases.

## Materials and methods

### Sample collection

To elucidate the evolution of GAstVs, 208 sequences were obtained from the National Center for Biotechnology Information (NCBI, https://www.ncbi.nlm.nih.gov) on May 1, 2025. Currently, GAstV has only been reported in China, with no documented cases in other countries worldwide; therefore, all viral sequences were exclusively obtained from Chinese sources. Eight duplicate sequences and seventeen unverified GAstV sequences were removed from the dataset. The final dataset comprised 183 sequences.

### Phylogenetic analyses

Initially, we employed the Turkey astrovirus (GenBank accession No. NC002470) sequence as outgroup. Subsequently, we constructed a maximum likelihood (ML) phylogenetic tree using IQ-Tree v.1.6.5, adopting the optimal evolutionary model recommended by the program after 1000 bootstrap replicates. The tree was rooted with the Turkey astrovirus related to GAstV, and long branches were pruned to enhance the resolution of the studied viruses. Nucleotide sequences were aligned using MAFFT v.7.402, and the resulting phylogenetic tree was visualized with TVBOT (https://www.chiplot.online/tvbot.html).

### Recombinant analyses

We screened the GAstV sequence dataset for recombination using RDP, GENECONV, Chimaera, axChi, and 3Seq. A second round of recombination analysis was conducted using BootScan and SiScan within the Recombination Detection Program version 5 (RDP5). Only recombination events validated by at least four methods were subjected to further evaluation of recombination probability, with an acceptable significance threshold of *p* < 0.05.

### Comparison of capsid protein sequences from two groups

The 183 amino acid sequences of ORF2 were analyzed using the meta-data-driven comparative analysis tool (meta-CATS), with a significance threshold set at a p-value of 0.05. This threshold represents the maximum probability level at which differences between groups could be attributed to chance, allowing for the identification of significantly distinct sites across the six subgroups.

## Results and discussion

### Geographical and temporal analysis of GAstVs

**Fig. 1:**
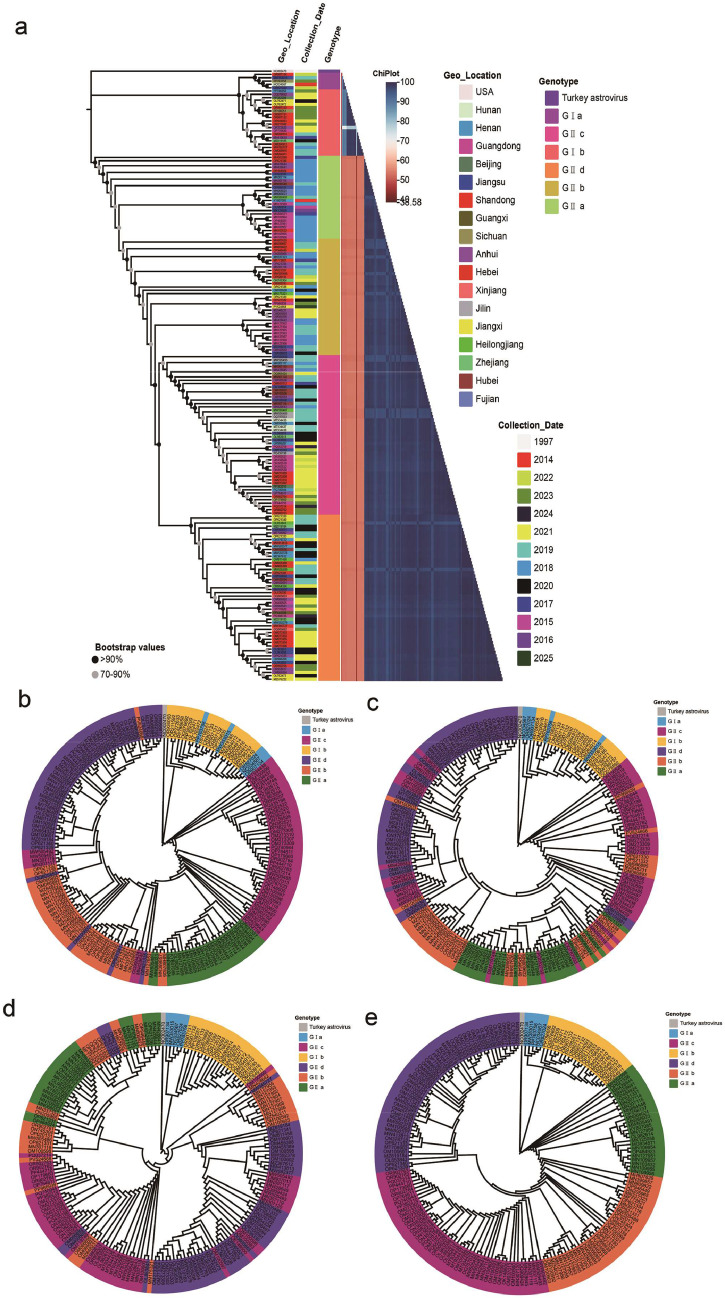
**Genotyping and phylogenetic analysis of GAstV strains based on whole-genome and different gene regions.** (a) A maximum-likelihood phylogenetic tree (with 1,000 bootstrap replicates) constructed from whole-genome sequences, illustrating the genotypic classification and geographical origins of 183 GAstV strains. (b–e) Maximum likelihood phylogenetic trees constructed using (b) ORF1a, (c) ORF1b, (d) ORF2 gene regions, and (e) complete genome sequences (1,000 bootstrap replicates). The GenBank accession numbers, and subgroups of each strain are annotated.

The GeneBank currently contains 208 complete GAstV genome sequences, with the earliest dating back to 2014. Over the past decade, these isolates have been detected in 17 Chinese regions, underscoring the virus's expanding epidemiological footprint. After removing unverified and redundant entries, a total of 183 sequences were ultimately obtained. Temporal distribution analysis revealed that 141 sequences (accounting for 77.05%) were predominantly derived from the period between 2018 and 2021. The significant increase in uploaded genome sequences during this period may be attributed to the rising infection rates and the increasing recognition of the importance of active monitoring (Fig. 1a).

The results of sequence sampling analysis indicate that most of the sequences were uploaded from Guangdong and Shandong provinces, which are major goose-farming regions. However, it is worth noting that GAstV has recently been discovered in Xinjiang. Regarding host sources, only two sequences lacked host information. Notably, one sequence was derived from a layer chicken (Wei et al., 2023), two sequence originated from Cherry Valley ducklings (Chen et al., 2020), while the remaining sequences originated from goose.

### Phylogenetic analyses of ORF1b, ORF1b, ORF2 genes, and complete genomes of GAstV

Phylogenetic tree analysis (Fig. 1a) based on 183 complete GAstV genome sequences demonstrated that GAstV can be distinctly classified into two groups, GI and GⅡ, consistent with previous research findings (Xiang et al., 2024). Further subtyping analysis revealed that GI could be subdivided into two subgroups (GI a and GI b), while GⅡ diverged into four subgroups (GⅡ a, GⅡ b, GⅡ c, and GⅡ d). This complex subgroup differentiation pattern may reflect the combined effects of multiple selective pressures during viral evolution, including host adaptation, geographic environmental factors, and viral mutation rates.

Notably, the SDHZ strain (GenBank No.: OP946449) isolated from chicken hosts was identified as a member of the GⅡ b subgroup. Homology comparison results demonstrated substantial genetic divergence between GI and GⅡ group (nucleotide identity <80%), while high homology (>90%) was maintained within each genotype. These genetic variation characteristics suggest that GAstV possesses strong continuous evolutionary capacity, with evolutionary drivers potentially originating from host immune pressure and environmental selection factors.

To elucidate the evolutionary mechanisms of GAstV, the phylogenetic trees were constructed based on the ORF1a, ORF1b, ORF2, and complete genome sequences of 183 GAstV sequences, respectively. The results demonstrated markedly distinct evolutionary histories among these three genes and the whole genome (Fig. 1b–e). Within the GⅡ group, the phylogenetic topology of the ORF1a gene exhibited the closest resemblance to that of the complete genome, whereas the ORF1b and ORF2 genes displayed more intricate evolutionary relationships. This phylogenetic incongruence was characterized by well-defined anomalous branching among subgroups, which was further confirmed by recombination analysis to stem from genomic recombination events. In contrast, within the GI group, the phylogenetic pattern of the ORF2 gene showed high similarity to that of the complete genome. These findings not only substantiate that intra-clade recombination facilitates genetic exchange among distinct subgroups, thereby expanding the genetic diversity of co-circulating GAstV strains, but also suggest that the ORF1b and ORF2 genes may serve as "hotspot" regions for genomic recombination, playing a pivotal role in the adaptive evolution of the virus.

### Recombination analysis of GAstVs

The recombination analysis results revealed that 27 potential recombination events have contributed to the genetic diversity of current GAstV isolates (Table 1). Within the GI group, four GAstV strains (C102, C1357, G2332, and ZJX14) were identified as products of recombination involving seven GAstV isolates. The dominant parental strains, FLX/2014 (GenBank No.: NC034567.1) and JSXZ/2022 (GenBank No.: OR827024.1), both belong to the GI a subgroup. The GII group exhibited more active recombination characteristics, with 23 recombinant strains identified, including GII a (2 strains), GII b (7 strains), GII c (11 strains), and GII d (3 strains). Notably, the GII a subgroup served as the major parental strain in 11 recombination events, suggesting its central role in GAstV evolution.

**Table 1:** Identification of 27 potential recombination events in the complete genomes of GAstVs in China during 2014–2025 using RDP5 software package.

Recombination Events serial numbei	Recombinant		Major parent		Minor parent		Detection methods						
	GenBank ID: Virus name/Year	Geno-group	GenBank ID: Virus name/Year	Geno-group	GenBank ID: Virus name/Year	Geno-group	R	G	B	M	C	S	T
1	OL982614.1: JSCZ15/2015	GⅡ a	MZ648231.1: ZM/2021	GⅡ b	OM719749.1: JSTH/2022	GⅡ c	+	+	+	+	+	+	+
2	MZ648231.1: ZM/2021	GⅡ b	MN307114.1: HN01/2018	GⅡ a	OQ805852.1: XJHT-1/2023	GⅡ d	+	+	+	+	+	+	+
3	OP621332.1: SD04/2020	GⅡ b	OR906207.1: HNxY2106/2021	GⅡ c	OQ805852.1: XJHT-1/2023	GⅡ d	+	+	+	+	+	+	+
4	OP621340.1 JX03/2019	GⅡ d	OM811458.1: ZYL01/2018	GⅡ d	OP621330.1: JX04/2021	GⅡ b	+	+	+	+	+	+	+
5	MN307116.1 AH02/2018	GⅡ b	OP946449.1: SDHZ/2022	GⅡ b	MN307119.1: HB02/2019	GⅡ c	+	+	+	+	+	+	+
6	OM066895.1: ZY02/2021	GⅡ b	MN307114.1: HN01/2018	GⅡ a	OM273302.1: G512/2021	GⅡ d	+	+	+	+	+	+	+
7	MT934437.1: HNU-LYG2/2019	GⅡ c	MN307114.1: HN01/2018	GⅡ a	OQ805852.1: XJHT-1/2023	GⅡ d	+	+	+	+	+	+	+
8	PP966939.1: GZ2301	GⅡ b	MN307114.1: HN01/2018	GⅡ a	OQ805852.1: XJHT-1/2023	GⅡ d	+	+	+	+	+	+	+
9	OQ787033.1: JL01/2019	GⅡ c	OR906207.1: HNxY2106/2021	GⅡ c	MW505491.1: HLJYC/2019	GⅡ a	+	+	+	+	+	+	+
10	OP621330.1: JX04/2021	GⅡ b	OR906207.1: HNxy2106/2021	GⅡ c	OQ805852.1: XJHT-1/2023	GⅡ d	-	+	+	+	+	+	+
11	OR906207.1: HNxy2106/2021	GⅡ c	MN307114.1: HN01/2018	GⅡ a	OM273302.1: G512/2021	GⅡ d	+	+	+	+	+	-	+
12	PP382210.1 J2/2021	GⅡ c	OM100600.1: JS01/2020	GⅡ c	OR906208.1: HNxy2111/2021	GⅡ d	+	+	+	+	+	+	+
13	OM100600.1: JS01/2020	GⅡ c	MN307114.1: HN01/2018	GⅡ a	OM273302.1: G512/2021	GⅡ d	+	+	+	+	+	+	+
14	OM273307.1: G529/2021	GⅡ c	MN307114.1: HN01/2018	GⅡ a	OM273302.1: G512/2021	GⅡ d	+	+	+	+	+	+	+
15	OR906208.1: HNxy2111/2021	GⅡ d	MW5S2379.1: HNXX-6/2020	GⅡ d	OP621334.1 AH01/2019	GⅡ b	+	+	+	+	+	+	+
16	OP621335.1: AH02/2021	GⅡ d	MW5S2379.1: HNXX-6/2020	GⅡ d	OP621332.1: SD04/2020	GⅡ b	-	-	-	+	+	+	+
17	MG934571.1 GD/2017	GⅡ a	OM719749.1: JSTH/2022	GⅡ c	MZ648231.1: ZM/2021	GⅡ b	+	-	-	+	+	-	-
18	OM719749.1: JSTH/2022	GⅡ c	OL982613.1: XSSH/2020	GⅡ c	MG934571.1 GD/2017	GⅡ a	+	+	+	+	+	+	+
19	OL982615.1: JSZ29/2020	GⅡ c	MN307114.1: HN01/2018	GⅡ a	OQ805852.1: XJHT-1/2023	GⅡ d	+	+	+	+	+	+	+
20	OL982613.1: XSSH/2020	GⅡ c	MN307114.1: HN01/2018	GⅡ a	OM273302.1: G512/2021	GⅡ d	+	+	+	+	+	+	+
21	OK148600.1: SQ/2020	GⅡ c	MN307114.1: HN01/2018	GⅡ a	OM273302.1: G512/2021	GⅡ d	+	+	+	+	+	+	+
22	PV524668.1: JXNC1/2025	GⅡ b	OL762473.1: JXGZ/2020	GⅡ d	OR902763.1: U56/2023	GⅡ c	+	+	+	+	+	+	+
23	OR902763.1: U56/2023	GⅡ c	MN307114.1: HN01/2018	GⅡ a	OQ805852.1: XJHT-1/2023	GⅡ d	+	+	+	+	+	+	+
24	PP763758.1: C102/2023	GⅠ a	NC034567.1: FLX/2014	GⅠ a	OR907133.1 C1357/2023	GⅠ b	+	+	+	+	+	+	+
25	OR907133.1 C1357/2023	GⅠ b	OR827024.1: JSXZ/2022	GⅠ a	OR907134.1: G2332/2022	GⅠ a	+	+	+	+	+	+	+
26	OR907134.1: G2332/2022	GⅠ a	NC034567.1: FLX/2014	GⅠ a	MH410610.1: AHDY/2017	GⅠ b	+	+	-	+	+	+	+
27	MZ819185.1: ZJC14/2020	GⅠ b	OR827024.1: JSXZ/2022	GⅠ a	OR907134.1: G2332/2022	GⅠ a	+	+	+	-	+	+	+

Breakpoint localization analysis demonstrated that among the 27 recombination events, five breakpoints were entirely located within the ORF1a gene region, four were confined to the ORF2 gene region, and the remaining breakpoints were distributed across intermediate regions of ORF1b and the ORF2 gene. Recombination not only enhanced the genomic diversity of GII strains but also generated novel evolutionary groups (classified as the GII c subgroup) through genetic exchange between GII a and GII d strains. The emergence of these novel recombinant strains has increased the genetic complexity and heterogeneity of field strains; however, their infectivity characteristics and pathogenic mechanisms require further investigation.

### Analysis of amino acid sequences of complete capsid protein

The variation of the capsid protein is significant for understanding the genetic relatedness of GAstV strains. To elucidate amino acid differences between the two genetic groups, we conducted mutational analysis on the capsid protein sequences of 183 GAstV sequences. A total of 210 amino acid sites exhibiting significant variations between the two groups were successfully identified. Additionally, seven distinct amino acid sites were found to differentiate GI a from its six genetic subgropus (R74K, V94I, E369S, E421G, Q497T, I597T, and S640D). Further amino acid variation analysis was performed separately for the GI and GII genetic groups. Using the FLX strain as a reference, the GI group exhibited 85 amino acid mutation patterns. Notably, two amino acid deletions (RD) at positions 652-653 and one deletion (F) at position 706 were observed, serving as distinguishing features between the GI a and GI b subgroups. In contrast to the GI group, the GII group demonstrated remarkable conservation in the capsid protein, with only 11 characteristic mutations identified among different genetic subgroups.

To further elucidate the molecular characteristics of viruses from different host origins, we conducted a systematic comparative analysis of the ORF2 amino acid sequences between chicken-origin SDHZ strain, duck-origin SDXT strain (MN399857), and goose-origin reference strains. The results revealed that the chicken-origin SDHZ strain harbored three specific amino acid variations in the capsid protein (R51K, K388N, and D570N), which differed from both the duck-origin SDXT strain and other goose-origin genetic subgroups. Notably, the duck-origin SDXT strain itself carried two unique amino acid (A249S and A295D) variation sites. These host-specific amino acid variations may be involved in regulating viral host adaptation, although their precise mechanisms in host range expansion remain to be elucidated. Subsequent studies are recommended to employ reverse genetics techniques for constructing recombinant viruses to systematically evaluate the impact of these key amino acid sites on host tropism determination. This study represents the first report of specific molecular markers in the capsid protein among avian-origin isolates from different hosts, providing important clues for understanding the cross-species transmission mechanisms of this virus.

## Funding

This work was supported by the Liangshan Prefecture Science and Technology Bureau with support from Ningbo (Grant No. 24NBYJ0002).

## Disclosures

The authors declare that they have no known competing financial interests or personal relationships that could have appeared to influence the work reported in this paper.
